# Content Validity Index and Intra- and Inter-Rater Reliability of a New Muscle Strength/Endurance Test Battery for Swedish Soldiers

**DOI:** 10.1371/journal.pone.0132185

**Published:** 2015-07-15

**Authors:** Helena Larsson, Matthias Tegern, Andreas Monnier, Jörgen Skoglund, Charlotte Helander, Emelie Persson, Christer Malm, Lisbet Broman, Ulrika Aasa

**Affiliations:** 1 Department of Neurobiology, Care Sciences and Society, Division of Physiotherapy,Karolinska Institutet, SE-141 83, Huddinge, Sweden; 2 Swedish Armed Forces, Stockholm, SE-107 85, Sweden; 3 Department of community medicine and rehabilitation, Umeå University, SE-901 87, Umeå, Sweden; 4 Department of Surgery and Perioperative Science, Umeå University, SE-908 87, Umeå, Sweden; Duke University, UNITED STATES

## Abstract

The objective of this study was to examine the content validity of commonly used muscle performance tests in military personnel and to investigate the reliability of a proposed test battery. For the content validity investigation, thirty selected tests were those described in the literature and/or commonly used in the Nordic and North Atlantic Treaty Organization (NATO) countries. Nine selected experts rated, on a four-point Likert scale, the relevance of these tests in relation to five different work tasks: lifting, carrying equipment on the body or in the hands, climbing, and digging. Thereafter, a content validity index (CVI) was calculated for each work task. The result showed excellent CVI (≥0.78) for sixteen tests, which comprised of one or more of the military work tasks. Three of the tests; the functional lower-limb loading test (the Ranger test), dead-lift with kettlebells, and back extension, showed excellent content validity for four of the work tasks. For the development of a new muscle strength/endurance test battery, these three tests were further supplemented with two other tests, namely, the chins and side-bridge test. The inter-rater reliability was high (intraclass correlation coefficient, ICC_2,1_ 0.99) for all five tests. The intra-rater reliability was good to high (ICC_3,1_ 0.82–0.96) with an acceptable standard error of mean (SEM), except for the side-bridge test (SEM%>15). Thus, the final suggested test battery for a valid and reliable evaluation of soldiers’ muscle performance comprised the following four tests; the Ranger test, dead-lift with kettlebells, chins, and back extension test. The criterion-related validity of the test battery should be further evaluated for soldiers exposed to varying physical workload.

## Introduction

Heavy physical work demands is a reality for soldiers during military missions, notwithstanding that certain military occupations are exposed to higher physical load than others [1]. If a discrepancy exists between the work requirements and the soldiers’ physical capacity, disorders and disability in the long run could be attributed to physiological and mechanical stress to which soldiers were exposed [2–4]. Hence, to assure that soldiers have sufficient physical work capacity to complete military work tasks (i.e. combat readiness), muscle strength- and endurance tests are commonly used during selection and regular testing procedures [2,5–8].

To ensure valid evaluation of muscle strength and endurance according to the job requirements, occupational relevance is crucial [9–13]. Commonly used test batteries for soldiers have, however, in recent years, been challenged due to its lack of relevance [5–7]. For example, different versions of the Army Physical Fitness Test that only uses the soldiers own body weight when performing sit-ups and push-ups [5] may be considered inappropriate, since soldiers’ work tasks further require them to carry external loads [6,9,12,14]. Although research has shown that data obtained from physical tests can be used independently to predict future injury occurrence [2,15–17] or physical performance [14,18–20], the content validity of these tests are often not reported.

By definition, validity refers to a test or instrument measuring what it intends to measure [21]. There are, however, different categories of validity: face, content, construct and criterion. Face validity represents the person’s assumption and acceptance that a test represents the domain being assessed [22]. Even though face validity is a scientifically inferior method, it serves an important purpose, since tests or instruments without established face validity may not be relevant [21]. Like face validity, content validity is based on subjective judgement. That is, an expert’s opinion concerning a test that appears to serve the intended purpose [21,22]. To obtain content validity, the underlying construct of a test or instrument must be clearly conceptualised and clear evidence of the operational components must be defined [23]. Validation is often achieved by a panel of experts who review the proposed tests and determine its relevance in relation to the content domain. One proportion agreement method, the Content Validity Index (CVI), quantitatively estimates the content validity [22,23]. Previously, the method was described for determining and quantifying the validity of different items in questionnaires [22,23]. To control for high proportion of agreement due to random chance, modified Kappa statistics and calculation of the probability of chance are recommended [23]. When setting up test batteries, estimating the CVI is an important step for the selection of available and valid tests to further undergo criterion and construct validation [22,23].

Reliability sets the limits of validity of any test [21]. For muscle strength- and endurance tests to be reliable, the tests must yield similar results upon repeated administration [24,25]. In clinical practice, reliability is commonly determined based on measurements of the same subjects on two occasions, so called test-retest reliability (i.e. intra-rater reliability) [26,27]. Reliability is also dependent on multiple factors. The testing procedure being one, [21,24] where the reliability between raters (i.e. inter-rater reliability) is essential, since military testing must be executed on site at several different locations.

Usually, test batteries for soldiers consist of an assessment of whole-body endurance (aerobic capacity) and muscle endurance (repeated submaximal contractions, dynamic muscle endurance as well as isometric muscle endurance), and muscle strength and/or power [6,8].

The present study aimed to examine the content validity of commonly used muscle strength and endurance tests designed to evaluate/predict performance of physically demanding work tasks in military service. Further, it aimed to investigate the reliability of a proposed test battery.

## Methods

### Experimental Approach to the Problem

In the first part of the study, a consensus panel consisting of five Swedish Armed Forces employees with broad experience in both research and practical physical testing procedures identified the domain of content for essential job requirements, and defined the tests through discussions and a review of the literature [22]. In accordance with both the Swedish Armed Forces official policy [6] and as described in a technical report titled “Optimizing Operational Physical Fitness” from the North Atlantic Treaty Organization (NATO), the common physically demanding military work tasks for solders in modern fighting areas are marching, digging and material handling [8,28]. The conceptual definition of the dimension assessed in this framework was limited to investigating the content validity in the context of evaluating an individual’s basic condition [3]. The topic of “military work tasks” was divided into; (1) lifting (i.e. material handling), (2) carrying (walking with equipment attached to one’s body while marching as one would in a moving troop), (3) carrying (walking while holding an object in one’s hands), (4) climbing (e.g. crossing barriers when fighting in urban areas) and (5) digging. These tasks correspond unanimously to the duties of all soldiers during their first 12 weeks of basic military training in the Swedish Armed Forces, regardless of their future military occupational speciality (MOS).

After the three stages of the content validity evaluations, which comprised (1) the development stage, (2) the expert judgement stage and (3) the quantification stage (as further described below), the consensus panel proposed a test battery, that took into account the relevance of the varying physical demands of soldiers in the Swedish Armed Forces. The selected tests were investigated regarding its inter- and intra-rater reliability.

#### Ethics Statement

The regional Ethics Committee in Stockholm, Sweden, approved the study (Dnr: 2012/1690-32). All participants were given both verbal and written information, including a statement allowing the withdrawal of participation in the study at any time, prior to certifying the informed consent form.

### Content Validity Procedures

The Development Stage: Following a review of the literature, thirty physical performance tests were selected for examination by the consensus panel (see in APPENDIX, the Tests), and a rating protocol was designed. The selected tests, which are commonly used in the Nordic as well as the NATO countries, have been previously described in scientific and technical reports [6,8] and research publications [2,6]. However, the following tests, bench-press, loaded squat, shoulder press, isometric chest press, leg press and dead-lift with kettlebells were not explicitly described in any military testing publications up until the review conducted for this study (personal communication, Armed Forces in Norway and the Netherlands).

Eleven tests primarily assessed dynamic muscle endurance. The instructions were to perform maximum numbers of repetitions of push-ups [6], sit-ups in three different positions (crook lying, fixed feet and feet on bench) [3,6], pull-ups (chins) [6] and inclined chins [6], pull-ups (same as chins with reversed overhand grip), dips [6], lunges with 10 kg to 50 kg on the shoulders [6], dead-lift with kettlebells (see in “The standardized test protocol in the reliability investigation”), and the Ranger test, which is a load step test [2].

Fourteen tests primarily assessed muscle strength and/or power. These were vertical and horizontal jump [6], dead-lift with barbell [6], Isokai test (an isokinetic lift test) [29], incremental lift [8], box lift [8], bench press [30], chest press dynamic [8], chest press isometric (personal communication), shoulder press [30], loaded squat [30], leg press dynamic [8], leg press isometric (personal communication), and handgrip [8].

The remaining five tests evaluated isometric muscular endurance, namely side-bridge [6], plank with 0 kg to 20 kg weight [6], back extension [3], elbow-flex [8], and bent arm hang (arm suspension) [6].

The Expert Judgement Stage: The consensus group invited ten independent experts. The experts were chosen based on their experiences and well-informed knowledge of the discipline of muscle testing procedures in soldiers. In line with the recommendation by Lynn [22] stating a minimum of five and a maximum of ten experts to avoid possible random consensus [22,23] a total of nine experts accepted to participate. The experts represented Canada (n = 1), the Netherlands (n = 1) and Sweden (n = 7). Of them, six were medical experts (registered physiotherapists) of whom three held PhDs, including one professor, and two currently enrolled as PhD candidates. Three experts were military physical education teachers with MSc degrees, including one teacher working as a military officer for more than 15 years. A four-point ordinal Likert rating scale was used to evaluate the content validity of each of the 30 tests. The relevance of each test was assessed against the backdrop of the five military work tasks, specifically in terms of muscles involved, durations, movement patterns and loads lifted [1]. The nine experts rated the content validity of each test in relation to the five tasks in the rating protocol. The scale was scored accordingly: 1 = test not being relevant; 2 = somewhat relevant; 3 = quite relevant and; 4 = highly relevant. Grades 3 and 4 were considered acceptable [22]. Results from the nine experts´ judgements were quantified in the Quantification Stage.

### Procedures for Inter- and Intra-Rater Reliability of the Test Battery

#### Study design and subjects

An inter-rater reliability investigation of five of the tests, as identified based on the CVI evaluation (the three highest ranked tests supplemented by two commonly used military tests to ensure that domains such as upper-limbs and core-stability were explicitly covered), was performed with four raters that simultaneously measured the same group of subjects. The raters were physiotherapists who had more than seven years of clinical experience and were very familiar with testing procedures. At the time of data gathering, two of them were working in the Swedish Armed Forces. In total, 37 healthy engineer soldiers (33 men and 4 women) volunteered to participate (Table 1).

**Table 1:** Demographic characteristics of engineer soldiers, n = 37 (33 male and 4 female) in the inter-rater reliability, and ranger soldiers, n = 20 (male soldiers) in the intra-rater reliability (test-retest) investigation.

	Engineer soldiers			Ranger soldiers		
	Mean	(SD)	Min-Max	Mean	(SD)	Min-Max
Age (year)	26	(6)	19–46	24	(2)	20–31
Height (m)	1.80	(0.08)	1.63–1.98	1.84	(0.05)	1.78–1.95
Weight (kg)	81	(13)	56–118	87	(9)	73–110
BMI (kg/m^2^)	24.7	(2.4)	21–32	25.8	(2.6)	22–35

SD = standard deviation, BMI = body mass index.

An intra-rater reliability study of all five tests was carried out on two separate occasions seven days apart on ranger soldiers (n = 20) [31]. During the second test, the raters were blinded to the results of the first test. Demographic characteristics are presented in Table 1.

#### The standardized test protocol in the reliability investigation

Dead-lift with kettlebells: Pairs of kettlebells with a weight of 24 kg, 32 kg and 40 kg each were placed in three lines on the floor. The total weight to be lifted is thereby 48 kg, 64 kg and 80 kg, respectively. Subjects were instructed to perform two lifts on each, starting with the two lower weights, then progress to the heaviest weight. When necessary, (for subjects with short calf muscles) a 2 cm high board was placed under the heels. With knees bent, subjects securely held the two 40 kg kettlebells, followed by lifting as many times possible in one minute by fully straightening their legs while maintaining a straight back and eyes directed forward. The number of correctly performed lifts was counted.

Side-bridge [6]: The subjects laid sideways with the elbow and foot of the lower side of their body in contact with the floor, while maintaining the upper arm and leg along the side of the body. As described in detail by Malmberg [6] the subjects looked ahead and kept their body in a completely straight position, with the head and spine aligned with the legs, for as long as possible (s).

Chins [6]: The subjects hung from a chin up bar using an overhand grip, hands placed shoulder wide apart, legs extended and feet raised from the ground. They lifted their body until the chin reached a level over the bar and thereafter lowered the body until elbows were fully extended, similarly as described in detail by Malmberg [6]. The number of lifts correctly performed until failure was counted.

Back extension [3]: Subjects laid prone on a bench with the ankles fixed, the anterior iliac spines on the edge of the bench and the upper body unsupported. The subjects kept the upper body in a horizontal position as long time as possible (s), with flexed arms, hands at the level of the ears, and the elbows straight out from their bodies, as described in detail by Larsson [3].

The Ranger test, (a lower-limb functional capacity test) [2]: Wearing a 20 kg backpack, the subjects stood with the left foot on a 0.40 m high bench and performed a step up with the right foot. The period when both feet were on the bench, the body was straightened (during every repetition). One leg was tested at a time, 75 repetitions needed to be fulfilled at a rate of 25 steps per minute [2]. (The standard procedure for ranger soldiers is performing 75 steps with each leg loading with a 20 kg backpack).

### Statistical Analyses

Statistical analyses were conducted using IBM SPSS Statistics 22 (IBM Corporation, USA).

For establishing content validity, the CVI was calculated by dividing the number of experts that arrived at an acceptable test grade 3 (quite relevant) or 4 (highly relevant), by the total number of assessments of the test [22,23]. The cut-off for an excellent level was >0.78 for the tests rated by nine experts, that is, at least seven out of the nine experts agree [23]. (If all experts are in agreement the CVI = 1.0 and when eight out of nine agree the CVI = 0.89).

A modified Kappa value was calculated, the Kappa designating agreement of relevance, using the formula: (k* = (CVI- *P_c_*)/(1- *P_c_*) [23]. The probability of chance occurrence (*P_c_*) was computed using the formula for a binominal random variable, with one specific outcome: *P_c_* = [*N*!/*A*! *(N–A*)!].5^N^ where *N* = number of experts and *A* = Number agreeing on good relevance [23]! = a mathematical symbol for the product of all positive interfere less than or equal to N, for example 5! to mean 5x4x3x2x1. Guidelines to evaluate the relevance of the tests were applied using an evaluation criteria for considering values for Kappa, as proposed by Cicchetti and Sparrow: Fair = k* of 0.40 to 0.59; Good = k* of 0.60 to 0.74; and Excellent = k* of 0.75 to 1.00 [32].

#### Inter- and intra-rater reliability

Initially, to check for systematic bias, outliers or heteroscedasticity data were visualised using Bland-Altman plots [27].

Relative reliability was assessed by calculating the intraclass correlation coefficient, (ICC_2.1_) where the following formula was used: (BMS-EMS)/(BMS+(k-1)EMS+*k*(JMS-EMS)/*n*). For intra-rater reliability, the model ICC_3.1_ was used according to the formula: (BMS-EMS)/(BMS+(k-1)EMS). As per the formula, BMS is the between-subjects mean square, EMS is the residual (error) mean square, JMS is the between- subjects mean square, *n* the number of subjects and *k* the number of raters or tests [33]. Assessment of ICC statistics was conducted using the criteria described by Currier with reliability coefficients categorised as follow; 0.90 to 0.99 = high, 0.80 to 0.89 = good, 0.70 to 0.79 = fair and ≤ 0.69 rated as poor [34].

The standard error of the mean (SEM) is the square root of the total mean square error (WMS) from the repeated measures analysis of variance (ANOVA) which evaluates the absolute reliability and is expressed in the same units of measurement as the original scores SEM = √WMS [24,25,27]. The SEM %, the within-subject standard deviation as a percentage of the mean (i.e. independent from the units of measurement) was also calculated as SEM% = (SEM/mean)x100 [24,25,27]. The smallest real difference (SRD) was used to evaluate clinically important changes, meaning the smallest measurement change that can be interpreted as a real difference. This was calculated as follow: SRD = √2x1.96xSEM [24,25,27].

## Results

### Content Validity

The CVI of the muscle tests for carrying, lifting, digging and climbing is shown in Table 2. The tests are presented in order of the level of agreement and numbers of tasks judged as ‘excellent’. Sixteen of the tests had a CVI ≥0.78 for representing one or more of the military tasks. Three of them (as for the Ranger test, dead-lift with kettlebells and back extension) had excellent content validity, which are relevant to 4 of the 5 tasks. Fourteen of the tests were considered valid for measuring lifting tasks. Five of these reached a CVI of 1.00, demonstrating complete agreement among experts. In the present investigation, fourteen of the reviewed tests as listed in Table 2 were not considered valid for any of the military tasks.

**Table 2:** The content validity index (CVI) and the Kappa designating agreement of relevance (k*) of the muscle tests for carrying, lifting, digging and climbing.

	Carry (on the body)		Carry (with hands)		Lifting		Digging		Climbing	
Test	CVI	k*	CVI	k*	CVI	k*	CVI	k*	CVI	k*
***Lower-limb tests***										
**Ranger test**	**1.00**	1.00	**0.89**	0.89	**0.89**	0.89	0.67	0.60	**0.89**	0.89
Lunges (10–50 kg)	**0.89**	0.89	**0.78**	0.76	**0.89**	0.89	0.56	0.41	0.67	0.60
Loaded squat	0.67	0.60	0.67	0.60	**1.00**	1.00	0.67	0.60	**0.89**	0.89
Leg press, dyn.	0.56	0.41	0.67	0.60	**1.00**	1.00	0.67	0.60	0.67	0.60
***Lift tests***										
**Dead-lift with kettlebells**	**0.78**	0.76	**0.89**	0.89	**1.00**	1.00	**0.89**	0.89	0.67	0.60
Isokai test	0.67	0.60	**0.89**	0.89	**1.00**	1.00	**0.89**	0.89	0.67	0.60
Dead-lift	0.67	0.60	**0.89**	0.89	**1.00**	1.00	**0.89**	0.89	0.56	0.41
Incremental lift	0.44	0.26	**0.89**	0.89	**1.00**	1.00	0.67	0.60	0.33	0.20
Box-lift	0.33	0.20	**0.78**	0.76	**1.00**	1.00	0.56	0.41	0.33	0.20
***Trunk tests***										
**Back extension**	**0.78**	0.76	**0.78**	0.76	**0.78**	0.76	**0.78**	0.76	0.22	0.16
**Side-bridge**	0.67	0.60	0.56	0.41	**0.78**	0.76	0.67	0.60	0.56	0.41
Plank	**0.78**	0.76	0.67	0.60	0.67	0.60	0.67	0.60	0.44	0.26
***Upper-limb tests***										
**Chins**	0.22	0.16	0.33	0.20	**0.78**	0.76	0.44	0.26	**0.89**	0.89
Pull-ups	0.11	0.10	0.44	0.26	**0.78**	0.76	0.67	0.60	**0.78**	0.76
Inclined chins	0.22	0.16	0.22	0.16	0.67	0.60	0.44	0.26	**0.78**	0.76
Hand grip	0.00	0.00	**0.78**	0.76	**0.78**	0.76	0.67	0.60	0.67	0.60

Tests scoring a CVI of ≥ 0.78 were equivalent to a probability of a chance occurrence (*P_c_*) of <0.07, indicating an excellent level of the expert agreement concerning the tests’ relevance. (All figures are presented in S2 Table: Results Content Validity Index.)

CVI ≥ 0.78 is considered an excellent level, as marked in the table with bold figures. Tests listed below did not reach the proportion of experts whose endorsement was required to establish content validity beyond the level of significance in any of the evaluated tasks; leg press isometric, horizontal jump and vertical jump, sit-ups, sit-ups 90° in hips, sit-ups fixed feet, push-ups, bent arm hang, shoulder press, elbow-flex, dips, chest press dynamic, bench press, chest press isometric.

### Inter- and Intra-Rater Reliability

The results of the inter-rater reliability investigation, with four raters and 37 subjects, found the reliability to be high for all tests in the test battery (i.e. the Ranger test, dead-lift with kettlebells, back extension, chins and side-bridge test). The relative reliability, assessed with the intra-class correlation ICC_2,1,_ was 0.99 (95% confidence interval (CI) ranging between 0.989 to 1.0). The absolute reliability SEM of the four tests ranging from 0.3 to 1 (ranging between <1 repetition to 4 repetitions and 1s to 3 s) and SEM% between 0.9 to 5.5, thus reflecting small dispersion of the measurement errors between raters.

The intra-rater reliability investigation included 20 subjects. Mean and SD for each test and occasion are presented in Table 3.

**Table 3:** Test performance of the intra-rater reliability part of the study.

		Test 1		Test 2	
Test	n	Mean	(SD)	Mean	(SD)
Dead-lift kettlebells 80 kg, (reps)	20	21	(7)	22	(7)
Side-bridge, left (s)	19	100	(33)	113	(40)
Side-bridge, right (s)	19	110	(37)	123	(46)
Chins (reps)	20	10	(6)	10	(5)
Back extension (s)	20	98	(27)	103	(31)
Ranger test 20 kg, left (75 reps)	20	73	(9)	73	(7)
Ranger test 20 kg, right (75 reps)	20	73	(9)	74	(5)

n = number of subjects, s = seconds, reps = number of repetitions.

**Fig 1:**
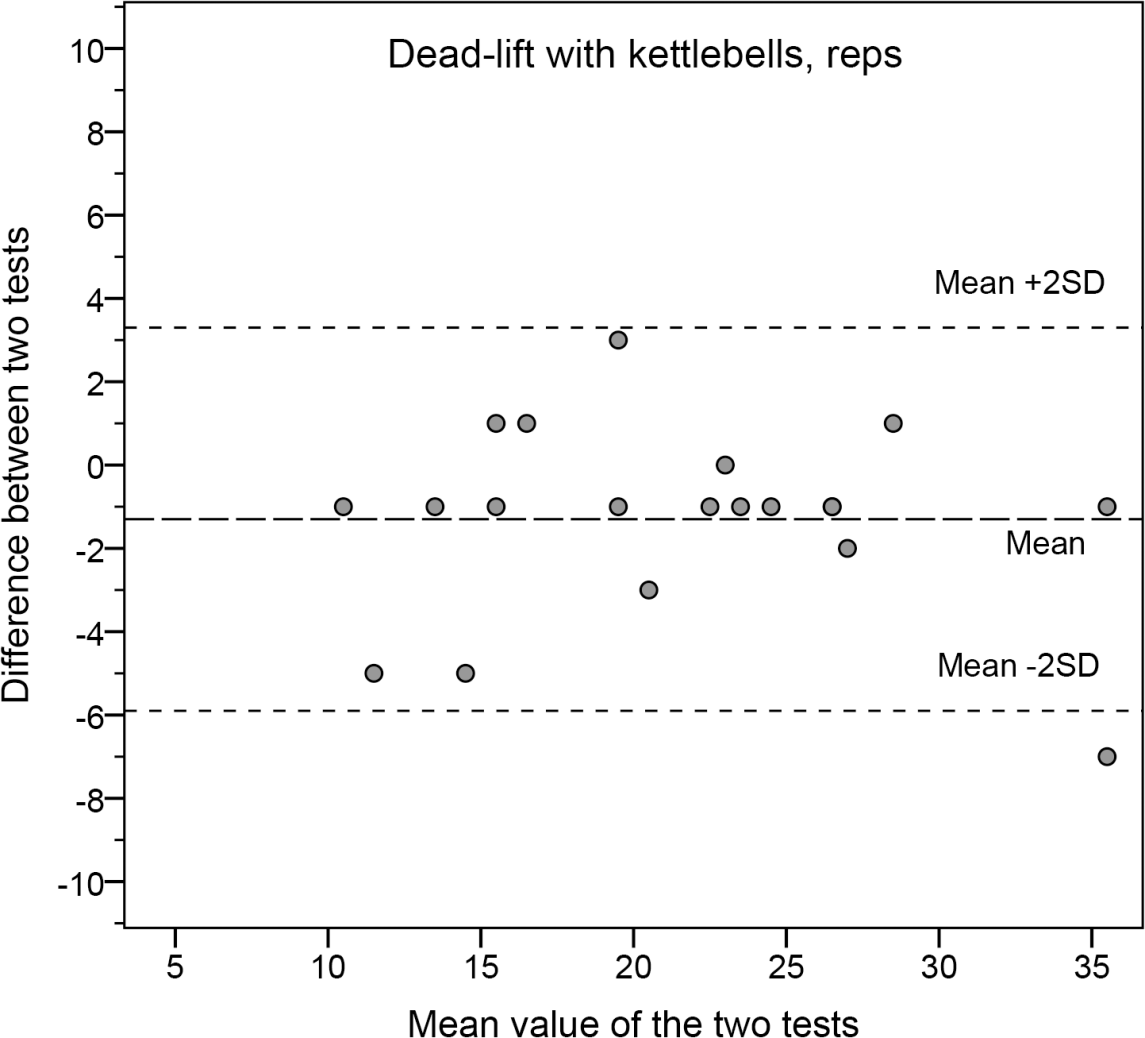
The differences between Test 1 and Test 2 for dead-lift with kettlebells.

**Fig 2:**
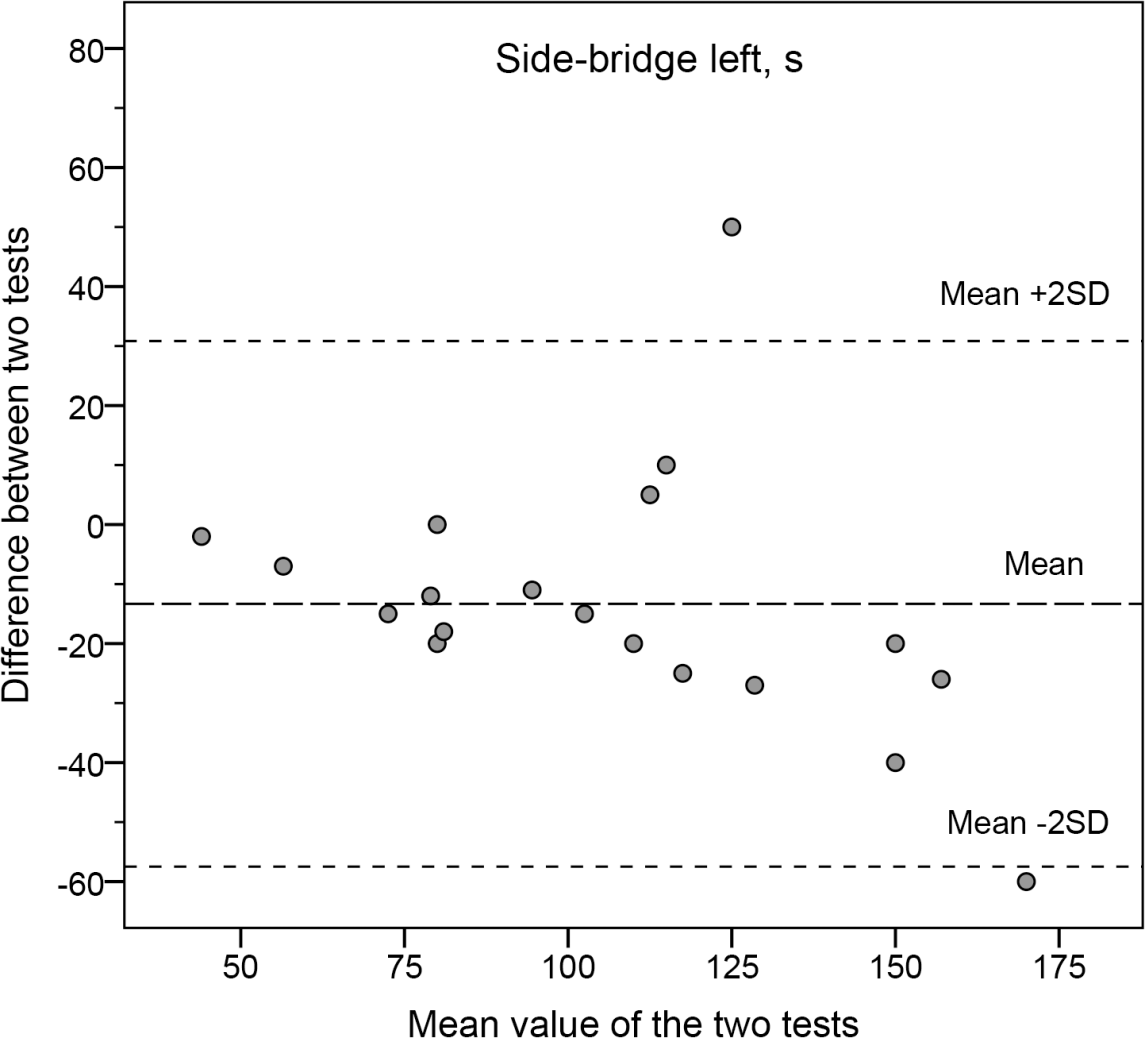
The differences between Test 1 and Test 2 for the side-bridge test.

As seen in Figs 1 and 2 the Bland-Altman plots represent tests with high and low reliability, respectively.

Bland-Altman plots illustrate the mean of the two test occasions (x-axis) and the mean differences between the two occasions (y-axis) for each subject. Dashed lines illustrate the mean and ±2SD for the group (y-axis).

The relative intra-rater reliability found good to high ICC_3,1_ for all five tests. The absolute intra-rater reliability measured as SEM, was acceptable with the exception of the side-bridge with a SEM% value >15%, Table 4.

**Table 4:** Intra-rater reliability of the tests.

Test	n	Mean	ICC_3.1_ (95%CI)	SEM	SEM %	SRD
Dead-lift kettlebells 80 kg, (reps)	20	21	0.95 (0.88–0.98)	2	8.5	5
Side-bridge, left (s)	19	107	0.82 (0.59–0.93)	18	16.8	50
Side-bridge, right (s)	19	116	0.91 (0.78–0.96)	15	13.2	42
Chins (reps)	20	10	0.94 (0.86–0.98)	1	14.2	4
Back extension (s)	20	100	0.85 (0.66–0.94)	11	11	32
Ranger test 20 kg, left (75 reps)	20	73	0.97 (0.92–0.99)	1	2	4
Ranger test 20 kg, right (75 reps)	20	73	0.88 (0.73–0.95)	3	3	7

n = number of subjects, s = seconds, reps = number of repetitions, ICC_3,1_ = intraclass correlation coefficient, 95%CI = 95% confidence interval, SEM = standard error of measurement, SEM% = standard error of measurement in per cent, SRD = the smallest real difference.

### Suggested Strength/Endurance Test Battery

After the content validity evaluation and the intra-rater reliability investigation, the consensus panel proposed the following tests to be included in the new test battery: the Ranger test, dead-lift with kettlebells, chins, and back extension test. The reason for choosing these tests was based on the relevance thereof for soldiers in physically demanding units and due to the high absolute and relative inter-rater reliability of tests.

## Discussion

The main finding of this study was that 16 out of 30 evaluated muscle performance tests were considered content valid for testing soldiers’ physical capacity according to work requirements. The five tests included in the investigated test battery (the Ranger test, dead-lift with kettlebells, chins, back extension and side-bridge tests) were found to have excellent content validity and high absolute and relative inter-rater reliability. While these tests also showed *good* and *high* intra-rater relative reliability, the absolute reliability of the side-bridge test was less acceptable.

Low content validity was rated in 14 commonly used physical performance tests. These results are in accordance with earlier findings suggesting that general physical fitness has shown to be a poor predictor of job performance [5,9,13,14,35]. For example, push-ups and sit-ups which are the most frequently occurring military tests in many countries, including Sweden [6] and the United States [5,36], were not considered to be content valid in the present work. As discussed in the consensus group and in earlier studies, these tests might favour subjects with low body weight. This is in contrast to field requirements where high body weight, in terms of more muscle mass, is necessary for load-carrying capacity [9,12]. This finding is important since the use of invalid tests could affect selection of personnel, implementation of specific exercise training programs and evaluations. As such, it could have a negative effect on work task success and health among military personnel. In addition, invalid tests may also be discriminatory, since personnel are selected based on such arbitrary criteria.

Three tests were considered relevant for four of the five evaluated tasks (i.e. “the Ranger test”, dead-lift with kettlebells lift and back extension). Together, they cover all domains being evaluated; lifting, carrying-walking with equipment attached to one’s body, carrying-walking while holding an object in one’s hands, climbing and digging. In addition, the consensus panel supplemented the test battery with the side-bridge and chins tests. The reason for including chins was to admit a test that placed high demands on the shoulders while lifting the body [6], for example, when soldiers have to enter buildings or cross barriers. Notably, chins were selected over pull-ups after discussion in the consensus group. The overhand grip was considered more relevant in different military situations.

The results of the present study are in agreement with earlier findings on the relevance of a load-carrying test and a lift-test in the evaluation of soldiers’ physical performance [11,12]. Soldiers are heavily loaded, with a total weight of 55 kg worn on the body while marching, also commonly seen during exercise in the Swedish Armed Forces and by U.S. Ranger soldiers during the war in Kuwait and Afghanistan [3,35,37]. The previously evaluated “Ranger test” that measures the capacity of carrying equipment on one’s body with the loaded step-test is used in the selection process of ranger soldiers in the Swedish Armed Forces. This test was found to predict the premature discharge of conscripts from military service [2]. Furthermore, the weight increases when soldiers are required to wear supplies and/or hold weapons in their hands. The dead-lift with kettlebells test that supplements the Ranger test, measure one’s capacity of lifting and carrying while holding an object in one’s hands. The pattern of movement during a kettlebells lift is similar to material handling, lifting boxes, cans and stretchers, etc. However, it is important to remember that the loads differ depending on the unit as well as the soldiers’ military function [35]. Thus, the amount of external exposure needs to be further evaluated in a more objective validity investigation, that is, through criterion-related or construct validation of actual tasks [22,23]. For example, the weight of the kettlebells as well as the weight of the backpack is easily adjustable when testing soldiers for specific military work tasks and different MOS.

Accordingly, it can be questioned whether isometric tests with no external load, such as the back extension and the side-bridge tests [6], are relevant for measuring performance of specific military work tasks. The experts were not in total agreement on the content validity, and a possible reason for the back extension test to achieve an excellent CVI could be that the test evaluates the ability of the spine and hip to extend as these muscle groups act against gravity in order to keep the body upright. Contrary to many other muscle groups, the erector spinae muscle needs to work isometrically. The importance of the ability to maintain spinal alignment while performing different military tasks was also discussed in the consensus group. Another reason for including this test was earlier findings indicating that a lack of strength correlated with pain in the lower back (i.e. shorter maximal contraction times and functional disability) [17,38,39].

Reliability sets the limit of validity [21], meaning a test could not be valid if it lacks reliability. The inter-rater reliability, as assessed by 4 raters and 37 subjects in a single trial, was high for all five tests (ICC_2,1_ = 0.99), with a small dispersion of the measurement errors between raters. The small variation between raters established the consistency of the test battery, indicating the administration of the test battery by different raters. The intra-rater reliability was good to high (ICC_3,1_ 0.82–0.97) for the five tests, according to the criteria described by Currier [34]. In contrast to a study by Evans et al [10], the findings of the SEM% independent of the units of measurements used (i.e. the assessment of absolute reliability) must be discussed, especially for the side-bridge test. The SEM% for the side-bridge (left side) was 17%, with an SRD of 50 seconds. This could indicate that the test lack standardisation, and it was biased due to a low sample size. The sample size recommendations are usually based on practical experience. Commonly, a sample size of 15–20 is used in studies with continuous data, but larger sample sizes (30–50) have been suggested for more useful SRDs [24].

When judging the relevance of different tests and quantifying the results, it is important to evaluate different aspects of the CVI method. For example, aspects such as the operationalization of the underlying constructs, the selected experts, bias arising from the experts themselves as well as the inadequate specification to the experts [23] should be formally noted or assessed. We acknowledge that the ratings could have differed if other experts were chosen. In the present study, the selection of a panel of experts was carefully made in order to ensure high-level competency concerning the subject matter. The types of work tasks vary within the armed forces, but the most common physical work tasks for soldiers, at least within NATO countries and in the Swedish Armed Forces, include marching, climbing, digging and material handling [6,8,28]. Material handling involves different kinds of lifts and material transfer; carrying equipment attached to one’s body and carrying objects in one’s hands [3,40]. While test batteries used in military services consisted of both central and peripheral performance tests, the present investigation was limited to muscle endurance (repeated submaximal contractions i.e. dynamic muscle endurance as well as isometric muscle endurance) and muscle strength and/ or power.

In our on-going work with the development and evaluation of a valid test battery for selection of personnel and for the evaluation of an exercise-training program, the CVI assessment can be considered the first critical step. The next step will be to investigate the criterion-related validity of this test battery designed for the evaluation of different physiological demands required to complete specific job tasks included in military work.

## Conclusion

A new test battery for soldiers is presented and it comprises of a functional loaded step test (the Ranger test), dead-lift with kettlebells, chins, and the back extension test. Excellent content validity and good to high inter- and intra-rater reliability were found for all included tests. The test battery should be further evaluated for criterion-related validity for soldiers exposed to physically demanding tasks.

## Supporting Information

S1 Table Appendix, the Tests. (PDF)

S2 Table Results Content Validity Index. (PDF)
