# Supplementary material for: Content Validity Index and Intra- and Inter-Rater Reliability of a New Muscle Strength/Endurance Test Battery for Swedish Soldiers
Source: PLoS One. 2015 Jul 15;10(7):e0132185. doi: 10.1371/journal.pone.0132185 (PMC4503674; doi:10.1371/journal.pone.0132185)
Supplement: S1 Table — (PDF) [file pone.0132185.s001.pdf]

APPENDIX, the Tests

1.	*Push-ups* (dynamic muscle endurance)<br>The test person adopts the support-standing position with elbows and arms fully stretched and a shoulder width between hands. The body shall maintain a straight line from head to knees. The person lowers the body to a point where the upper arms are parallel to the ground, and then presses the body up to the starting position. Maximal numbers of repetitions (Larsson 2009).	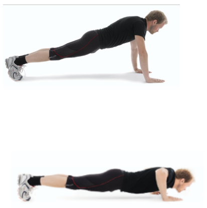
2.	*Sit-ups* (dynamic muscle endurance)<br>Sit down with a 90° angle in knee joint and arms coupled together. Look forwards and keep feet in contact with the floor. Lower trunk until lower back touches floor and then back to starting position. Maximal numbers of repetitions (Malmberg 2011).	
3.	*Sit-ups with fixed feet* (dynamic muscle endurance)<br>According to the APFT instructions. Lie on the back with knees bent at a 90° angle and fixed feet, fingers interlocked behind the head. Raise the upper body to vertical, and then return to starting position. Numbers of successfully completed repetitions in one or two (APFT) minutes (Malmberg 2011).	
4.	*Sit-ups with hip and knee in a 90° angle* (dynamic muscle endurance)<br>Lie on back with knees and hips bent 90° and feet placed on a bench. Keep hands holding ears. Lift upper body, elbows touch thighs, and then return to starting position. Maximal numbers of repetitions, rate 25 per minute (Larsson 2009).	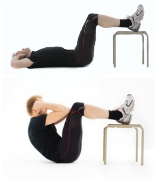
5.	*Side-bridge* (isometric muscle endurance)<br>Lie sideways. Support position by placing the elbow underneath shoulder. Keep the legs straight and the top arm placed along the body and head lifted, making a straight line from head to toes. Lift body, maintaining the straight, correct static position as long as possible. Perform test on each side once (Malmberg 2011).	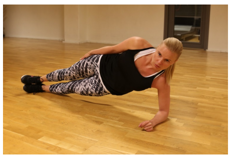
6.	*Plank with 0 kg to 20 kg weight* (isometric muscle endurance)<br>Lie face down and place elbows directly below shoulders and rest on forearms. Place toes on floor and lift body keeping a straight line from head to toes. Maintain position as long as possible. (From Danish Armed Forces Physical Tests - a 0-20 kg weight is placed over the loins) (Malmberg 2011).	
7.	*Back extension* (isometric muscle endurance)<br>Lie prone over a bench with ankle fixation and with upper body outside bench and fists placed near ears. Keep upper body horizontal and unsupported as long as possible (Larsson 2009).	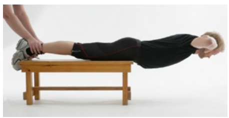

8.	*Pull-ups* (dynamic muscle endurance)<br>Grab the chin-bar with the hands shoulder-width apart in an underhand grip, palms facing towards you. Pull yourself up, cheek above the bar, and then lower yourself in a controlled fashion. Maximal number of repetitions (Larsson 2009).	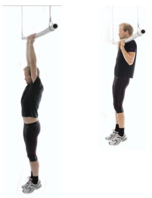
9.	*Chins* (dynamic muscle endurance)<br>Same as above (pull-ups) with reverse overhand grip. Maximal numbers of repetitions (Malmberg 2011).	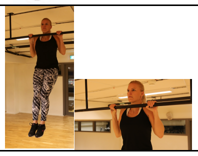
10.	*Inclined chins* (dynamic muscle endurance)<br>Hang horizontally from a bar and support feet on a bench with hands in overhand grip. Keep body straight. Pull upper body until chest touches bar. Maximal number of repetitions (Eisinger et al in NATO report, 2009).	
11.	*Dips* (dynamic muscle endurance)<br>Hang with straight arms in dips poles. Lower to position with 90° in the elbow joint and then return to starting position. Do as many correct repetitions as possible (Malmberg 2011).	
12	*Lunges with 10 kg to 50 kg* (dynamic muscle endurance)<br>The test is carried out as a fall-out with an alternating right and left leg, so that the heel of the front leg is a minimum of 10 cm in front of the knee of the back leg. Kneel with weights of 10 to 50 kg.<br>Knee of back leg must reach a distance at least 10 cm from the floor and remain stable during the test. The exercise is repeated continuously or to exhaustion (Malmberg 2011).	
13.	*Vertical jump* (assessing strength/power)<br>From an upright position bend down, swing both arms down and back, quickly swing both arms forward and up, and jump as high as possible. The height is defined as the distance between initial standing mark and the top of the jump (Malmberg 2011).	
14.	*Horizontal jump* (assessing strength/power)<br>With the legs side-by-side, make an active lowering to a 90° angle in knee joint, tilting upper body forward and swaying arms back. Turn into an explosive stretching of the body in a horizontal jump as far as possible. Distance is measured from start line to landing line (Malmberg 2011).	
15.	*Ranger test (Step-up)* (dynamic muscle endurance)<br>Stand with either foot on a bench, 0.4 m high, during the whole exercise. Grab the straps of the 20 kg backpack. The foot is placed on the bench. With the other foot, step up and down to the bench. Perform 75 repetitions on each side, step rate 25 per minute (Larsson 2006).<br>(Backpack weight 12 kg to 30 kg according to different trades).	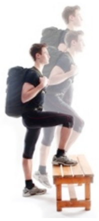

16.	*Dead-lift* (muscle strength, single or repetitive lifting)<br>A single or repetitive lifting measure.<br>Lower bum against heels. Grasp the rod with hands at shoulder width. Secure your back by keeping it straight, with a little sway. Perform a lift by straightening your legs (Malmberg 2011).	
17.	*Dead-lift kettlebells* (muscle strength, single or repetitive lifting)<br>Pairs of kettle bells with a weight of 16, 24, 32 and 40 kg. Lower bum against heels. Grasp one kettle bell in each hand, shoulder width apart. Secure the back. Perform lift by straightening legs. The kettle bells must touch the floor prior to each lift (personal communication).	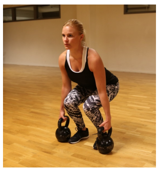
18.	Isokai test (isokinetic lift, muscle strength, a single lift measure)<br>Pull the bar vertically from the knees and up to the level of the chest by straightening legs into a full straight up position. The strength is measured in Newton, maximal strength and average of the lift, respectively (Larsson et al 2011).	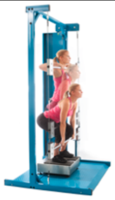
19.	*Incremental lift* (muscle strength, single or repetitive lifting)<br>The test is performed in an incremental lift machine. The lifting height and load based on the demands of the job (Brock and Legg 1997, Rayson et al 2000).	
20.	*Box lift* (muscle strength, single or repetitive lifting)<br>Lifting an ammunition box with side handles, with increasing load from ground to 1.70 m and to 1.45 m respectively. The score is the maximum load lifted (Rayson et al 2000).	
21.	*Handgrip* (muscle strength)<br>Strength of both hands measured using a dynamometer (Brock and Legg 1997, Bilzon et al 2002).	
22.	*Leg press* (isometric muscle strength)<br>Sit with feet placed on the platform linked to a power cell. Adjust seat to reach 120° angle in knee joint and grasp the handles. Make a maximal leg press and keep the position for about five sec. After one repetition a 45 -60 sec break is given before the next try (personal communication).	
23.	*Leg press* (dynamic muscle strength)<br>Sit with 90° angle in knee joint. Push the leg press device until reached fully stretched legs. The procedure is repeated three times and the best result is record (Eisinger et al in NATO report, 2009).	
24.	*Chest press (Seated bench press)* (isometric muscle strength)<br>Sit down and grasp the handles with a shoulder width grip with a 120° angle in elbow joint. Make a maximal chest –press and keep the position for about five sec. After one repetition a 45 -60 sec break is given before the next try (personal communication).	
25.	*Chest press (Seated bench press)* (dynamic muscle strength)<br>Sit down with hands placed in a broad wrist grip and feet flat on the floor. Press the bench press device away from the chest until reached arms fully stretched (Eisinger et al in NATO report, 2009).	

26.	*Elbow-flex* (isometric muscle endurance)<br>Standing with feet shoulder width apart and knees slightly flexed. Holding a box, weighing 14 kg, with flexed arms with the elbows by the side, the lower arm parallel to floor. Hold the static position as long as possible (Rayson et al 2000).	
27.	*Bent arm hang* (isometric muscle endurance)<br>Hang in a bar with bended arms, hands in an underhand grip, and shoulder width. The chin is positioned over the edge of the bar without touching the bar. Keep the static position as long as possible (Malmberg 2011).	
28.	*Bench press* (assessing strength/power)<br>Lay down on a flat bench. Grasp the bar with a 10 cm wider than shoulder width. Lower the bar to chest and turn the bar before it touches the chest. Press the bar up till elbows are being fully extended or slightly bended (personal communication).	
29.	*Shoulder press* (assessing strength/power)<br>Sit on a bench with back support. Grasp a weight in each hand. Press the weights up till fully extended arms or slightly bended. Return to starting position and repeat (personal communication).	
30.	*Loaded squat* (assessing strength/power)<br>Place a bar on the upper back. Stand stable with the feet placed hip width or wider. Bend knees and lower the bar. Turn the movement at 90° angle in knee and press the bar up to starting position (personal communication).	

Helena Larsson, PhD,
Department of Neurobiology, Care
Science and Society,
Karolinska Institutet, Sweden
and the Swedish Armed Forces
helena.larsson@mil.se

References:

Larsson H. Premature Discharge from Military Service -Risk Factors and Preventive Interventions (thesis) Stockholm 2009.
Malmberg. Physical Fitness in the Nordic Armed Forces. A description of Basic Test Protocols, 2011 Ed Malmberg J. Oslo.
NATO. *Optimizing Operational Physical Fitness.* 2009. France: North Atlantic Treaty Organisation.
Larsson H, Harms-Ringdahl K. A lower-limb functional capacity test for enlistment into SwedishArmed Forces ranger units. Mil Med. 2006 Nov;171(11):1065-70.
Larsson H, Tegern M, Broman L, Harms-Ringdahl K. Isokai-testet som urvalstest till utbildning i Försvarsmaktens nya personalförsörjningssystem. ISBN 978-91-7457-498-2. Stockholm (In Swedish).
Brock JR, Legg SJ. The effects of 6 weeks training on the physical fitness of female recruits to the British army. Ergonomics. 1997 Mar; 40(3):400-11.
Rayson, M., Holliman, D., & Belyavin, A. Development of physical selection procedures for the British Army. Phase 2: relationship between physical performance tests and criterion tasks. [Research Support, Non-U.S. Gov't]. *Ergonomics,* 2000 *43*(1), 73-105.
Bilzon JL, Scarpello EG, Bilzon E, Allsopp AJ. Generic task-related occupational requirements for Royal Naval personnel. Occup Med (Lond). 2002 Dec;52(8):503-10.
