# Supplementary material for: Content Validity Index and Intra- and Inter-Rater Reliability of a New Muscle Strength/Endurance Test Battery for Swedish Soldiers
Source: PLoS One. 2015 Jul 15;10(7):e0132185. doi: 10.1371/journal.pone.0132185 (PMC4503674; doi:10.1371/journal.pone.0132185)
Supplement: S2 Table — (PDF) [file pone.0132185.s002.pdf]

Results Content Validity Index

Test	n experts (9) in agreement	CVI a)	Pc b)	K* c)	Evaluation d)
**Push-ups**					
Carrying equipment	3	0.333	0.164	0.202	
Carrying hold	4	0.444	0.246	0.263	Fair
Lifting	5	0.556	0.246	0.410	Fair
Digging	4	0.444	0.246	0.263	Fair
Climbing	4	0.444	0.246	0.263	Fair
**Sit-ups, crook lying**					
Carrying equipment	1	0.111	0.018	0.095	
Carrying hold	1	0.111	0.018	0.095	
Lifting	2	0.222	0.070	0.163	
Digging	4	0.444	0.246	0.263	Fair
Climbing	4	0.444	0.246	0.263	Fair
**Sit-ups, fixed feet**					
Carrying equipment	1	0.111	0.018	0.095	
Carrying hold	1	0.111	0.018	0.095	
Lifting	1	0.111	0.018	0.095	
Digging	1	0.111	0.018	0.095	
Climbing	3	0.333	0.164	0.202	
**Sit-ups, feet on bench**					
Carrying equipment	1	0.111	0.018	0.095	
Carrying hold	1	0.111	0.018	0.095	
Lifting	2	0.222	0.070	0.163	
Digging	3	0.333	0.164	0.202	
Climbing	3	0.333	0.164	0.202	
**Side-bridge**					
Carrying equipment	6	0.667	0.164	0.601	Good
Carrying hold	5	0.556	0.246	0.410	Fair
Lifting	7	0.780	0.070	0.760	Excellent
Digging	6	0.667	0.164	0.601	Good
Climbing	5	0.556	0.246	0.410	Fair
**Plank with 0 kg to 20 kg weight**					
Carrying equipment	7	0.780	0.070	0.760	Excellent
Carrying hold	6	0.667	0.164	0.601	Good
Lifting	6	0.667	0.164	0.601	Good
Digging	6	0.667	0.164	0.601	Good
Climbing	4	0.444	0.246	0.263	Fair

Test	n experts (9) in agreement	CVI a)	Pc b)	K* c)	Evaluation d)
**Back extension**					
Carrying equipment	7	0.778	0.070	0.760	Excellent
Carrying hold	7	0.778	0.070	0.760	Excellent
Lifting	7	0.778	0.070	0.760	Excellent
Digging	7	0.778	0.070	0.760	Excellent
Climbing	2	0.222	0.070	0.163	
**Pull-ups**					
Carrying equipment	1	0.111	0.018	0.095	
Carrying hold	4	0.444	0.246	0.263	Fair
Lifting	7	0.778	0.070	0.760	Excellent
Digging	6	0.667	0.164	0.601	Good
Climbing	7	0.778	0.070	0.760	Excellent
**Chins**					
Carrying equipment	2	0.222	0.070	0.163	
Carrying hold	3	0.333	0.164	0.202	
Lifting	7	0.778	0.070	0.760	Excellent
Digging	4	0.444	0.246	0.263	Fair
Climbing	8	0.889	0.014	0.890	Excellent
**Inclined chins**					
Carrying equipment	2	0.222	0.070	0.163	
Carrying hold	2	0.222	0.070	0.163	
Lifting	6	0.667	0.164	0.601	Good
Digging	4	0.444	0.246	0.263	Fair
Climbing	7	0.778	0.070	0.760	Excellent
**Dips**					
Carrying equipment	0				
Carrying hold	0				
Lifting	2	0.222	0.070	0.163	
Digging	4	0.444	0.246	0.263	Fair
Climbing	4	0.444	0.246	0.263	Fair
**Lunges with 10 kg to 50 kg on the shoulders**					
Carrying equipment	8	0.889	0.014	0.890	Excellent
Carrying hold	7	0.778	0.070	0.760	Excellent
Lifting	8	0.889	0.014	0.890	Excellent
Digging	5	0.556	0.246	0.410	Fair
Climbing	6	0.667	0.164	0.601	Good

Test	n experts (9) in agreement	CVI [a)]	Pc [b)]	K* [c)]	Evaluation [d)]
**Vertical jump**					
Carrying equipment	2	0.222	0.070	0.163	
Carrying hold	1	0.111	0.018	0.095	
Lifting	5	0.556	0.246	0.410	Fair
Digging	2	0.222	0.070	0.163	
Climbing	3	0.333	0.164	0.202	
**Horizontal jump**					
Carrying equipment	2	0.222	0.070	0.163	
Carrying hold	1	0.111	0.018	0.095	
Lifting	2	0.222	0.070	0.163	
Digging	1	0.111	0.018	0.095	
Climbing	1	0.111	0.018	0.095	
**Ranger test**					
Carrying equipment	9	1.0	0.002	1.0	Excellent
Carrying hold	8	0.889	0.014	0.890	Excellent
Lifting	8	0.889	0.014	0.890	Excellent
Digging	6	0.667	0.164	0.601	Good
Climbing	8	0.889	0.014	0.890	Excellent
**Dead-lift with barbell**					
Carrying equipment	6	0.667	0.164	0.601	Good
Carrying hold	8	0.889	0.014	0.890	Excellent
Lifting	9	1.0	0.002	1.0	Excellent
Digging	8	0.889	0.014	0.890	Excellent
Climbing	5	0.556	0.246	0.410	Fair
**Dead-lift with kettlebells**					
Carrying equipment	7	0.778	0.070	0.760	Excellent
Carrying hold	8	0.889	0.014	0.890	Excellent
Lifting	9	1.0	0.002	1.0	Excellent
Digging	8	0.889	0.014	0.890	Excellent
Climbing	6	0.667	0.164	0.601	Good
**Isokai test**					
Carrying equipment	6	0.667	0.164	0.601	Good
Carrying hold	8	0.889	0.014	0.890	Excellent
Lifting	9	1.0	0.002	1.0	Excellent
Digging	8	0.889	0.014	0.890	Excellent
Climbing	6	0.667	0.164	0.601	Good

Test	n experts (9) in agreement	CVI a)	Pc b)	K* c)	Evaluation d)
**Incremental lift**					
Carrying equipment	4	0.444	0.246	0.263	Fair
Carrying hold	8	0.889	0.014	0.890	Excellent
Lifting	9	1.0	0.002	1.0	Excellent
Digging	6	0.667	0.164	0.601	Good
Climbing	3	0.333	0.164	0.202	
**Box lift**					
Carrying equipment	3	0.333	0.164	0.202	
Carrying hold	7	0.780	0.070	0.760	Excellent
Lifting	9	1.0	0.002	1.0	Excellent
Digging	5	0.556	0.246	0.410	Fair
Climbing	3	0.333	0.164	0.202	
**Handgrip**					
Carrying equipment	0				
Carrying hold	7	0.778	0.070	0.760	Excellent
Lifting	7	0.778	0.070	0.760	Excellent
Digging	6	0.667	0.164	0.601	Good
Climbing	6	0.667	0.164	0.601	Good
**Leg press, isometric**					
Carrying equipment	3	0.333	0.164	0.202	
Carrying hold	3	0.333	0.164	0.202	
Lifting	5	0.556	0.246	0.410	Fair
Digging	3	0.333	0.164	0.202	
Climbing	3	0.333	0.164	0.202	
**Leg press, dynamic**					
Carrying equipment	5	0.556	0.246	0.410	Fair
Carrying hold	6	0.667	0.164	0.601	Good
Lifting	9	1.0	0.002	1.0	Excellent
Digging	6	0.667	0.164	0.601	Good
Climbing	6	0.667	0.164	0.601	Good
**Chest press, isometric**					
Carrying equipment	1	0.111	0.018	0.095	
Carrying hold	1	0.111	0.018	0.095	
Lifting	1	0.111	0.018	0.095	
Digging	1	0.111	0.018	0.095	
Climbing	1	0.111	0.018	0.095	

Test	n experts (9) in agreement	CVI a)	Pc b)	K* c)	Evaluation d)
**Chest press, dynamic**					
Carrying equipment	1	0.111	0.018	0.095	
Carrying hold	1	0.111	0.018	0.095	
Lifting	3	0.333	0.164	0.202	
Digging	3	0.333	0.164	0.202	
Climbing	1	0.111	0.018	0.095	
**Elbow-flex**					
Carrying equipment	0				
Carrying hold	6	0.667	0.164	0.601	Good
Lifting	3	0.333	0.164	0.202	
Digging	3	0.333	0.164	0.202	
Climbing	1	0.111	0.018	0.095	
**Bent arm hang**					
Carrying equipment	1	0.111	0.018	0.095	
Carrying hold	5	0.556	0.246	0.410	Fair
Lifting	2	0.222	0.070	0.163	
Digging	2	0.222	0.070	0.163	
Climbing	5	0.556	0.246	0.410	Fair
**Bench press**					
Carrying equipment	1	0.111	0.018	0.095	
Carrying hold	1	0.111	0.018	0.095	
Lifting	3	0.333	0.164	0.202	
Digging	2	0.222	0.070	0.163	
Climbing	1	0.111	0.018	0.095	
**Shoulder press**					
Carrying equipment	1	0.111	0.018	0.095	
Carrying hold	2	0.222	0.070	0.163	
Lifting	5	0.556	0.246	0.410	Fair
Digging	3	0.333	0.164	0.202	
Climbing	2	0.222	0.070	0.163	
**Loaded squat**					
Carrying equipment	6	0.667	0.164	0.601	Good
Carrying hold	6	0.667	0.164	0.601	Good
Lifting	9	1.0	0.002	1.0	Excellent
Digging	6	0.667	0.164	0.601	Good
Climbing	8	0.889	0.014	0.890	Excellent

a) CVI, content validity index.
b) pc (probability of a chance occurrence) was computed using the formula for a binomial random variable, with one specific outcome: pc =[N!/A!(N-A)!] *.5N where N =number of experts and A=Number agreeing on good relevance.
c) k*=kappa designating agreement on relevance: k*=(CVI-pc)/(1-pc).
d) Evaluation criteria for kappa, using guidelines described in Cicchetti and Sparrow (1981) and Fleiss (1981): Fair=k of .40 to .59; Good=k of .60 to .74; and Excellent=k > .74.
